# The Success of Topical Treatment of Onychomycosis Seems to Be Influenced by Fungal Features

**DOI:** 10.1155/2021/5553634

**Published:** 2021-07-09

**Authors:** Vanessa Vasconcellos-Pontello, Flávia Franco Veiga, Marina Cristina Gadelha, Marielen Ribeiro, Melyssa Negri, Terezinha Inez Estivalet Svidzinski

**Affiliations:** Departamento de Análises Clínicas e Biomedicina, Centro de Ciências da Saúde, Universidade Estadual de Maringá (UEM), Avenida Colombo, 5790, Maringá, PR, CEP 87020-900, Brazil

## Abstract

**Aim:**

To evaluate the topical treatment of onychomycosis using a 10% hydroalcoholic propolis extract (PE) in two aleatorily chosen patients and analyze possible risk factors from hosts including some particularities of the isolated fungi that may justify the outcomes achieved.

**Materials and Methods:**

A topical treatment, with PE, was started in two cases of toe onychomycosis due to *T. rubrum*. The *in vitro* PE antifungal activity against these isolates was confirmed. Moreover, the ability of the fungi to infect the human nail was evaluated also in an *ex vivo* study, analyzed by histopathology.

**Results:**

Within four months, both patients showed evident improvement, but with different outcomes. The possible host-related risk factors justifying the poorer outcome in patient 1 include a longer duration time of onychomycosis (50 years). Some particularities in the *T. rubrum* strain isolated from this patient in relation to that found in patient 2 were observed: (1) the hypha morphology suggesting a major adaptation of the fungus to the host; (2) a 16 times greater propolis concentration was required *in vitro*; and (3) a faster ability to start a growth using the nail as the only nutritional source. Additionally, this isolate was more efficient in producing a biofilm on the nail surface.

**Conclusions:**

A partial clinical and complete mycological cure for the two patients was achieved after four months of PE daily use. Despite a complete recovery, a different outcome was observed between both cases. A more persistent onychomycosis, added to greater fungal potential to produce biofilm on the nail, seems to influence greatly the success of a topical treatment with PE.

## 1. Introduction

Onychomycosis is the most common nail pathology in the world [1] mainly affecting people aged over 60 years [2, 3] and significantly impacting the quality of life [4]. Despite its great relevance, the therapeutic options available for onychomycosis are limited, have few pharmacological alternatives, and are slow and costly. Moreover, cure rates for onychomycosis are often low and relapses are frequent [2]. Systemic terbinafine is considered the gold standard for onychomycosis treatment [5, 6]. According to some authors, the topical administration of drugs is preferred over the systemic route as it has fewer side effects [3, 7].

In this context, new topical compounds have been developed and tested, but cure rates are still less than desirable [8]. Thus, it is necessary to develop a safe topical treatment, which has few side effects, has good nail permeation capacity, and is effective against the main onychomycosis-causing microorganisms [9]. Recently, propolis has been considered among the new therapeutic options in the management of superficial fungal diseases including onychomycosis [10]. In fact, propolis has exhibited suitable results in this context, and the authors proved that ethanol propolis extract (PE) is a topical therapeutic option for onychomycosis, in a translational study from *in vitro* to the clinics. Antifungal activity against the planktonic cells and biofilm formed by *Trichophyton* spp. was clearly demonstrated, besides the low cytotoxicity and the capacity of PE to penetrate human nails. However, the performance of this product in patients needs to be further investigated since the results, although promising, were heterogeneous, providing from partial or complete cure to no improvement [11]. Thus, the objective of the current study was to evaluate the topical treatment of onychomycosis using PE in two aleatorily chosen new patients and analyze possible risk factors from hosts besides some particularities of the isolated fungi that may justify the outcomes achieved.

## 2. Materials and Methods

### 2.1. Ethical Aspects and Patients' History

This study was approved by the Ethics Committee of the State University of Maringa (approval number 1246516), according to resolution no. 466/12 of research involving human beings and with the Declaration of Helsinki under ethical principles for medical research involving human beings.

Patient 1 is a 69-year-old male with no known underlying disease; his medical history includes abnormal fasting glycemia and, therefore, did not receive medication. He has had nail injury in both feet compromising one hallux and the adjacent skin (*tinea pedis*) for approximately 50 years; his nail bed was thickened and associated with nail dystrophy. He had been treated with oral medication in the past but without clinical cure. Patient 2 was a 65-year-old female with chronic hypertension on continuous medication. She presented a toe-nail damage with keratosis and associated dystrophy for at least six years. This patient had been treated, in another service, with topical terbinafine but without improvement.

These patients were selected from a bigger project that included our previous publication [11]. Both gave their written consent for the treatment of onychomycosis only by a topical application of a 10% hydroalcoholic propolis extract, made in ethanol (PE), provided by the UEM school pharmacy.

### 2.2. Nail Sampling and Microbiological Analysis

After clinical evaluation by a dermatologist, nail samples with clinically suspected onychomycosis were collected by scraping. This material was sent to the Laboratory of Medical Mycology, UEM, for processing.

Mycological diagnosis was performed in two stages: direct mycological examination with 20% KOH and 0.5% Evans blue to clarify the material and observation of the sample through light microscopy. The samples were also spread on sabouraud dextrose agar (SDA) (Difco™ Detroit) and selective agar for pathogenic fungi (Difco™ Detroit), with nine inoculations in each type of culture medium. The cultures were incubated at 25°C for 30 days to allow fungus growth, with daily evaluation to verify the growth rate. The identification of each isolated fungus considered growth time, uniformity of the colonies grown, macro and micromorphology, and biochemical test results [12]. In both cases, *T. rubrum,* an anthropophilic dermatophyte fungus, was identified. The isolates were deposited in the Microbial Collections of the Paraná Network (Taxonline) at the Federal University of Paraná as *T. rubrum* CMRP2912 and *T. rubrum* CMRP2918, respectively.

### 2.3. Treatment Application and Follow-Up

The first orientation was made just after the laboratory conclusion when the treatment was started. The lesions in the affected area were treated topically, exclusively with two drops, twice a day of PE. Both patients were instructed to clean their nails with soap, water, and a brush daily and to polish the affected areas of the nails weekly.

The follow-up included monthly interviews besides nail samples collection being conducted 1 month and 4 months later aiming to evaluate the clinical and laboratory outcomes.

### 2.4. Propolis In Vitro Susceptibility Testing

Some laboratory tests were performed on the clinical isolates obtained from the two patients, *T. rubrum* CMRP2912 and *T. rubrum* CMRP2918, using the reference *T. rubrum* ATCC 40051 strain. Each test was performed in duplicate and in two independent experiments.

The *in vitro* susceptibility test with the PE used in the patients was performed by the microdilution broth method described previously [11]. The serial dilutions of PE ranged from 17,500.00 to 34.17 *μ*g/mL of total polyphenols present in PE. Three controls were included: positive (fungal inoculum + culture medium), negative (culture medium only), and alcohol (fungal inoculum + ethyl alcohol at a concentration equivalent to that in PE). All preparations were incubated at 37°C for 48 h to determine the MIC for PE against the fungal strains. To complement this result, the minimum fungicide concentration (MFC) was determined by placing aliquots of MIC test preparations (fungi + PE) on SDA plates.

### 2.5. The Nail Invasion by an Ex Vivo Study

The ability of each of the fungal isolates to invade the nail plate was evaluated using nails obtained from healthy adult volunteers. The nails were manually cut into fragments of similar size and then autoclaved at 121°C for 20 min. An inoculum of 1 × 10^7^ CFU/mL (colony forming units per milliliter) of each fungus was prepared, and 500 *µ*L of each suspension was incorporated in a proportion of 1 : 100 into the yeast nitrogen base without amino acids agar (YNB agar, BD Difco^TM^ Detroit) while it was still liquefied. This preparation did not contain any nutritional source. After agar solidification, sterilized healthy nail fragments were introduced. The cultures were incubated at 25°C and inspected daily for three weeks. The time needed for each isolate to grow was evaluated, and the macroscopic and microscopic aspects of the growing colonies were studied.

### 2.6. Histopathologic Analyses on the Fungal Growth into the Nail

The infected nail fragments were embedded in paraffin, and continuous 4 micrometer sections were made. They were placed on glass slides and analyzed by histopathology processing methods. The sections were stained with periodic acid–Schiff (PAS) and Grocott's methenamine silver (GMS) followed by the analysis by light microscopy in 100× and 400× magnifications over the entire length of the nail.

## 3. Results and Discussion

**Figure 1:**
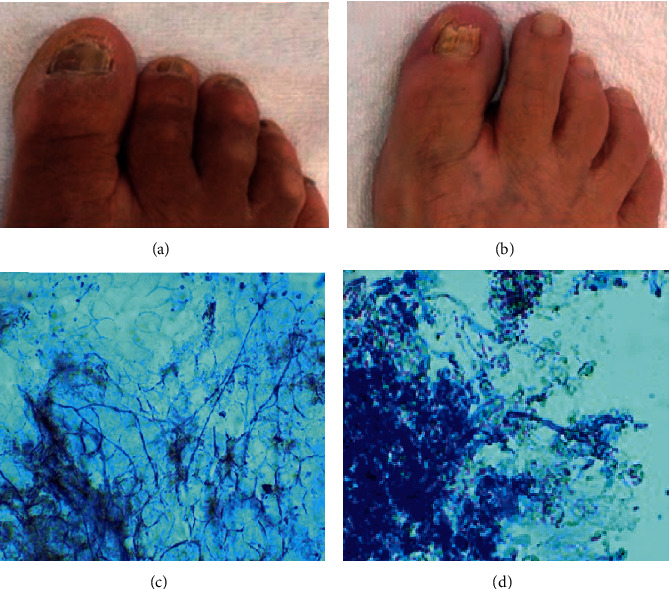
Clinical and laboratory findings (obtained through direct mycological examination) for onychomycosis in the two patients before treatment with propolis extract. Patient 1 (a) and (c). Patient 2 (b) and (d).

The treatment of onychomycosis is still a challenge [8] and needs to be improved since it significantly influences people's quality of life [4]. Studies have shown that the combination of topical and oral treatment provides the best results [13], but in some groups, such as older people, an effective topical treatment would be more interesting. The current research searched for some possible variables that could interfere with the outcome of a treatment of onychomycosis exclusively made with topical PE. The clinical and direct mycological findings for onychomycosis in the two patients before treatment with PE are shown in Figure 1.

**Figure 2:**
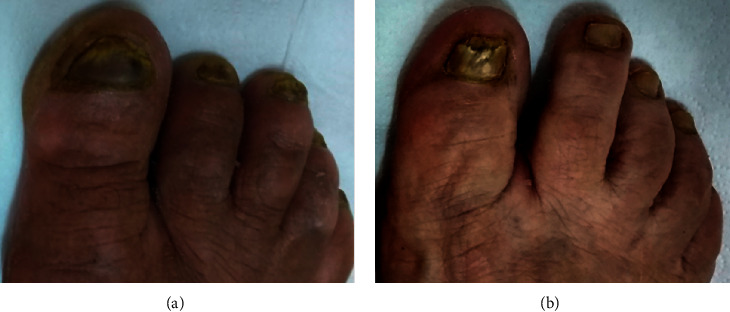
Clinical findings regarding the nails of the two patients after one month olf treatment.

**Figure 3:**
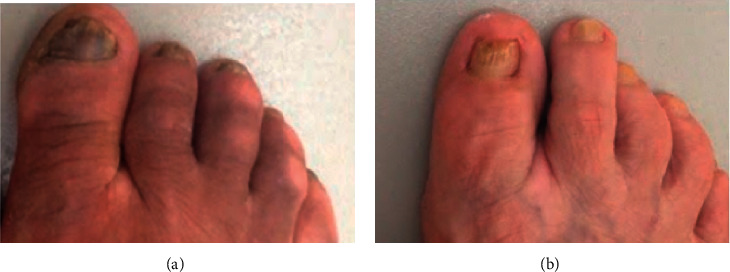
Nails treated with propolis extract for four months. (a) Patient 1 showed a clinical response of around 60%, and (b) patient 2 showed a better clinical response than patient 1, approximately 70%. Nail scrapings of both patients no longer showed the presence of fungal structures (indicating mycological cure).

After one month, none of the patients complained of discomfort with the medication (burning, erythema, or irritation). Only nail appearance was affected by a dark-yellowish color of the extract (Figure 2). However, little clinical improvement was observed, and upon direct mycological examination, many fungal structures were still observed in the lesion scrapings. After four months of treatment, there were mycological cure and partial clinical improvement of the nail lesions (approximately 60% and 70%, respectively) with apparent growth of a healthy underlying nail in both patients (Figure 3). These cure rates are close to those reported with synthetic drugs [5, 13, 14], and therefore, our results were considered promising.

Given the different clinical responses, we decided to look in depth searching among the characteristics of the two patients and their respective fungal isolates possible explanations for the initial therapeutic response. *T. rubrum* is the most frequent agent of onychomycosis [15]; it does not respond well to conventional antifungals [16]. It may cause both uncomplicated and complicated cases, including the dermatophytomas [17], making difficult in achieving satisfactory clinical treatment [6].

During the four months of follow-up, the two patients adhered to the treatment, but the response of patient 2 was better. Indeed, women usually achieve more success in the treatment of onychomycosis [3]. However, this study proposes to investigate other variables besides sex, which might result in different outcomes of treatment with PE.

Initially, patient 1 also presented *tinea pedis*, a known complication that may also influence onychomycosis cure [3]. This issue was resolved with the use of the same PE applied on the affected skin, resulting in complete resolution of *tinea pedis* (Figure 3). The too long duration of onychomycosis patient 1 (50 years) must also have negatively impacted the therapeutic response. Patient 2 also had onychomycosis for a long time (6 years), but significantly shorter. Although there are few reports in this regard, it is reasonable to imagine that lesions existing for such long durations are difficult to eradicate. In addition, patient 1, although not yet diagnosed with diabetes, had impaired fasting glycemia (prediabetic status), another risk factor associated with low therapeutic response of onychomycosis.

The current study investigated, for the first time, some possible intrinsic differences between the fungal isolates from the two patients, which could explain the differences found in the therapeutic outcomes.

First, differences in the morphology of the fungi were observed through direct mycological examination. Samples obtained through scrapings of the nails of patient 1 showed long, thin, regular hyphae in large quantities (Figure 1(c)); while, in samples from patient 2, there were shorter hyphae and in small quantities (Figure 1(d)). These differences in the fungal morphology could be considered as a result from different levels of adaptation of the fungus to the host, influencing the outcome of topical treatment. The hyphal morphology in patient 1 suggests a complete adaptation of the fungus to the host, with possible development of tolerance. In contrast, the hyphal morphology in material from patient 2 suggests a host resistance against the fungus, which would contribute to greater fungal vulnerability to topical medication.

**Figure 4:**
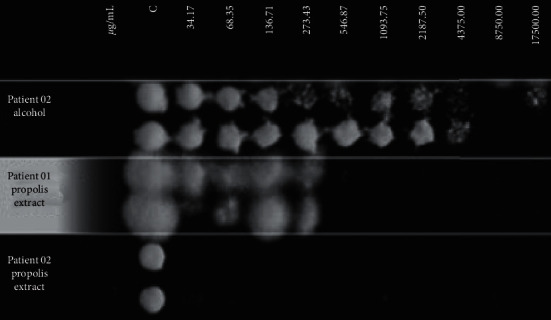
Minimum fungicidal concentration (MFC) of the propolis extract (PE) for *Trichophyton rubrum* isolated from two onychomycosis cases. Line 1 shows that ethanol (used as a propolis diluent) did not interfere with fungal growth. Line 2 shows the performance of PE against the isolate from patient 1 (MFC = 546.87 *μ*g/mL of total polyphenols present in PE). Line 3 shows the performance of the isolate from patient 2 (MFC = 34.17 *μ*g/mL of total polyphenols present in PE). C (control) = fungal inoculum without propolis or alcohol.

The *in vitro* susceptibility of fungal isolates to propolis also was investigated, as shown in Figure 4; the MFC of PE was 546.87 *μ*g/mL for the fungus isolated from patient 1 and 34.17 *μ*g/mL for the fungus from patient 2. Interestingly, the *in vitro* response of each strain was consistent with the respective performance by the each patient, i.e., the fungus found to be more sensitive *in vitro* was more responsive to therapeutic treatment. Therefore, these results confirm that these two fungal isolates had different responses to propolis, similarly to those found with classic antifungals [16].

**Figure 5:**
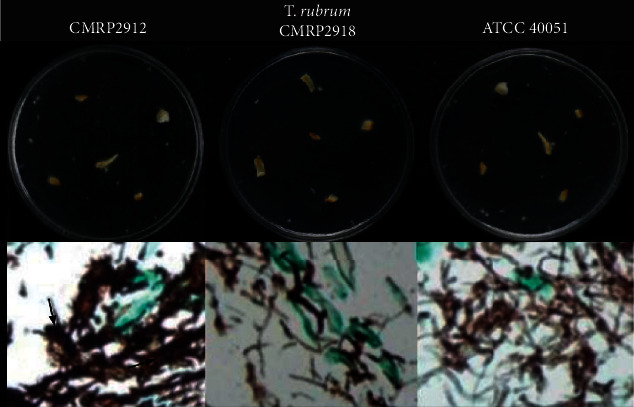
Illustration of the invasiveness of *Trichophyton rubrum* on a healthy human nail in ten days. CMRP2912: isolated from patient 1; CMRP2918: isolated from patient 2. *T. rubrum* ATCC 40051. Topline: macroscopic aspects of fungal growth in nail fragments. Bottom line: features of fungal invasion as seen under a light microscope. Histological sections of nails stained by GMS, at 400 ×.

Additionally, an *ex vivo* study was addressed aiming to better understand the interaction between the fungi and nail simulating a fungus/host relationship.  Figure 5 shows that, within 10 days, the three isolates (two clinical and one reference strain) started growing using the nail as the only nutritional source. Histological sections of the infected nails in this time, stained with GMS, confirmed the presence of hyphae inside the nails. *T. rubrum* isolated from patient 1 grew faster on the nail fragments and, interestingly, produced a component suggestive of an extracellular matrix (ECM) and therefore a supposed biofilm (arrow). Biofilm formation would probably be related to a dermatophytoma found in some patients [17].

This finding is fundamental to understand the complexity of case 1. Onychomycosis has been associated with the ability of fungi to naturally organize themselves into a biofilm [18] and produce an ECM, as suggested in Figure 5, which makes the permeation of antifungal drugs difficult. In this case, the longer onychomycosis time of patient 1 (50 years) could explain its more difficult eradication. Of the note, *T. rubrum* isolated from patient 2 (better outcome) was not able to produce biofilm on the nail in 10 days.

**Figure 6:**
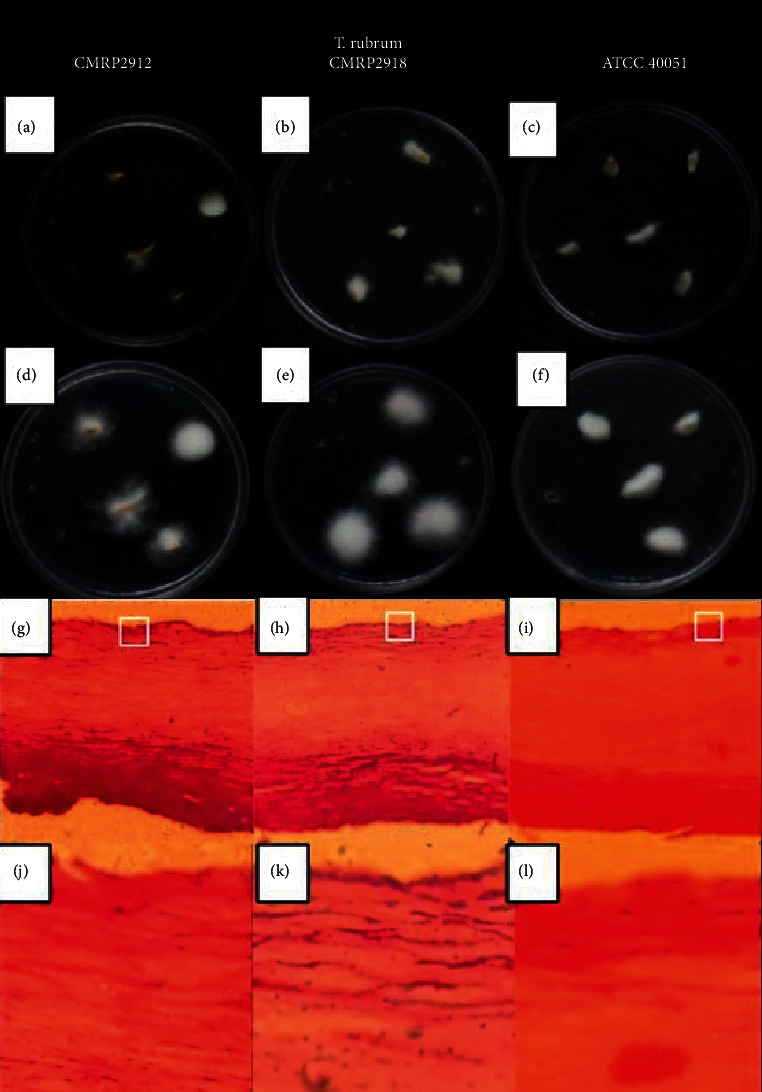
Macroscopic aspects of fungal growth on healthy nail fragments over two (a), (b), (c) and three weeks (d), (e), (f). Histological sections of *ex vivo* infected nails and PAS-stained nails, showing that hyphae penetrated the nails, at 400x (g), (h), (i) and 1000x (j), (k), (l).

Another issue would be the fungal ability to invade the nail; after three weeks of incubation, all fungi showed visible macroscopic growth on the nails. There was increased and consolidated mycelial growth with time (Figure 6). Histopathological analysis by PAS confirmed that all fungi penetrated the nail fragments and caused an *ex vivo* infection (Figures 6(g)–6(i)). It was also clear that the macroscopic growth, in the third week, was similar for the three strains evaluated. The results shown in Figures 5 and 6 allow us to conclude that the two fungal isolates have the ability to grow on the nail without another nutritional source. Furthermore, the fungus from patient 1 interacted more easily with the substrate (nail), probably explaining the lower therapeutic response.

Finally, despite all the factors associated with the poorer outcome of patient 1, it is important to highlight a significant improvement after four months of exclusively topical treatment with PE. In fact, the capacity of PE to act effectively on human pathogenic fungi [19, 20] has been previously confirmed for fungal biofilms [21, 22] and for the treatment of onychomycosis caused by opportunistic fungi [23].

## 4. Conclusions

This study confirmed the efficiency of a hydroalcoholic extract of propolis for the topical treatment of onychomycosis, without the need for facilitating vehicles. Mycological cure and partial clinical cure of the two patients with mild to moderate onychomycosis were observed after four months of daily use, twice a day.

The different performance of PE seems to be related to factors of the host such as lesion time and comorbidities. Moreover, it suggests the involvement of intrinsic factors related to the fungus as a greater ability to invade the nail and to form biofilm, besides natural different susceptibility to propolis.

## Data Availability

The data are available from the corresponding author upon reasonable request.
